# Non-invasive determination of disease activity in Crohn’s disease by serum luminex profiling

**DOI:** 10.1038/s41598-026-42925-x

**Published:** 2026-03-09

**Authors:** Gabriella A. Raffa, Regina N. Tyree, Kate Carson, Margaret M. Allaman, Dawn B. Beaulieu, Robin L. Dalal, Baldeep S. Pabla, Elizabeth A. Scoville, Sara N. Horst, David A. Schwartz, Mary K. Washington, Keith T. Wilson, Lori A. Coburn

**Affiliations:** 1 Division of Gastroenterology, Hepatology, and Nutrition, Department of Medicine, Vanderbilt University Medical Center, 2215B Garland Ave., 1030C MRB IV, Nashville, TN 37232 USA; 2 Department of Pathology, Microbiology and Immunology, Vanderbilt University Medical Center, Nashville, TN USA; 3 Center for Mucosal Inflammation and Cancer, Vanderbilt University Medical Center, Nashville, TN USA; 4 Veterans Affairs Tennessee Valley Health Care System, Nashville, TN USA

**Keywords:** Crohn’s disease, disease activity, inflammation, Luminex, biomarkers, Biomarkers, Diseases, Gastroenterology, Immunology, Medical research

## Abstract

Crohn’s disease (CD) is a type of inflammatory bowel disease (IBD), and treatment depends on disease activity assessment requiring invasive procedures. We aimed to identify serum cytokines/chemokines that differ between CD and controls, and by clinical, endoscopic, and histologic disease activity. Serum samples were obtained from 103 CD to 40 non-IBD controls undergoing colonoscopy. Clinical information was obtained by questionnaire and chart review. Disease activity was determined: clinically (Crohn’s Disease Activity Index), endoscopically (Simple Endoscopic Score for Crohn’s Disease), and histologically from colonoscopic biopsies. Cytokines/chemokines were measured by Luminex assay and compared by disease activity assessments (control vs. inactive vs. active), with adjustment for multiple comparisons. Correlation between activity indices and versus analytes were performed. Multiple analytes were significantly altered between clinically, endoscopically, and histologically inactive and active CD vs. control. One analyte, CXCL9, was significantly increased in active vs. inactive CD in endoscopic and histologic activity assessments. CXCL9 was the only analyte with a significant correlation to both SES (*r* = 0.57, q < 0.001) and histologic severity (*r* = 0.42, q < 0.001). CXCL9 had the highest discriminatory capacity for active vs. inactive disease both endoscopically (AUC = 0.76, 95% CI 0.65–0.87) and histologically (AUC = 0.79, 95% CI 0.70–0.88). Cytokine/chemokine profiling differentiated CD vs. controls and by disease activity, with CXCL9 as a potential marker distinguishing activity in endoscopic and histologic assessments. Serum cytokine/chemokine profiling may be a non-invasive strategy to assess disease activity in CD.

**Supplementary Information:**

The online version contains supplementary material available at 10.1038/s41598-026-42925-x.

## Introduction

Crohn’s disease (CD), a type of inflammatory bowel disease (IBD), is a chronic and debilitating condition with a complex pathogenesis. Inflammatory mediators, including pro-inflammatory cytokines, have been implicated in its disease course^1^. Over the years, new treatments have emerged, many of which target and modulate cytokine signaling pathways^2^. Induction and maintenance of remission are the cornerstones of management. Historically, clinical remission was the treatment target in CD. However, clinical symptoms do not always correlate with endoscopic or histologic appearance, risking persistent inflammation while the patient otherwise feels clinically well. Mucosal healing, as seen on endoscopic evaluation, has been shown to be prognostic of desired outcomes including clinical and steroid free remission^3^. Thus, the current treatment target in CD is symptom improvement and endoscopic healing, with histologic healing emerging as an important prognostic endpoint^4^. However, endoscopy is invasive and costly. In current clinical practice, biomarkers are used in conjunction with symptom monitoring (vs. symptoms alone), with a fecal calprotectin of > 150 µg/g and/or C-reactive protein (CRP) > 5 mg/L suggestive of ongoing inflammation^5^. However, CRP and fecal calprotectin are not specific for active CD^5^. Identifying more specific non-invasive markers of disease activity remains a strong area of interest.

Characterizing a patient’s disease state beyond the label of CD is paramount to personalized medicine. Studies have sought to further describe the inflammatory profiles of CD by disease location and activity, implicating that circulating serum factors can provide a snapshot into inflammatory processes including cytokines/chemokines and the acute phase protein leucine rich α-2 glycoprotein^6–8^. Knowledge of disease location has many useful applications, from treatment selection to prognostic information, as patients with ileal disease are more likely to experience disease-related complications, less likely to achieve remission, and in some studies, have less of a response to certain biologics^9^. Expression of cytokines and their receptors have been implicated as biomarkers for anti-TNFα therapy resistance^10–12^. Our group has previously demonstrated increased serum cytokines and chemokines in CD vs. non-IBD controls that correlated with clinical disease activity as determined by the Harvey Bradshaw Index using Luminex profiling^13^.

We hypothesize that serum cytokine and chemokine levels can distinguish CD from controls, and that serum inflammatory cytokine/chemokine profiling can be a non-invasive strategy for disease activity assessment in CD patients. This study aims to (1) identify serum cytokine and chemokine levels that differ between CD and controls, as well as by clinical, endoscopic, and histologic disease activity; and 2) to correlate disease activity assessments to each other and to serum analyte levels.

## Results

### Patient characteristics

In total, 103 CD patients and 40 non-IBD controls were included in the analysis. Patient characteristics are shown in Table 1. Between control and CD patients, age and body mass index (BMI) differed significantly. There were no differences in gender, race/ethnicity, tobacco smoking, or presence of major comorbidities (coronary artery disease, diabetes, or history of cancer) between the two groups. The majority of CD patients had ileocolonic disease (*n* = 94, 91.3%), as this was the focus for the original project, and were on disease-specific therapy (*n* = 86, 83.5%), with 38.8% being on anti-TNF-α therapy (*n* = 40). Characteristics of patients with clinically, endoscopically, and histologically inactive vs. active CD are shown in Supplemental Table S1. When comparing inactive and active groups by clinical, endoscopic, and histologic activity, there were no significant differences between age, gender, BMI, race/ethnicity, and tobacco use between groups. In comparison of clinically inactive vs. active CD, there were significantly more patients on immunomodulator therapy with clinically active CD vs. inactive CD (31.7% vs. 8.1%, *p* < 0.01); this difference was not significantly different in either histologic or endoscopic disease comparisons. Otherwise, there were no significant differences in disease phenotype or other IBD medication use (5-ASA, steroids, or biologics).

**Table 1:** Patient characteristics (control vs. CD).

	Control (*n* = 40)	CD (*n* = 103)
Age, median (25th − 75th percentile)	54.5 (50–60.3)	31 (25–47.5)***
Female Gender, n (%)	24 (60.0%)	63 (61.2%)
BMI, median (25th − 75th percentile)	28.1 (25–31.8)	26 (22–30.1)*
Race/Ethnicity, n (%)		
Asian, n (%)	1 (2.5%)	4 (3.9%)
Black or AA, n (%)	3 (7.5%)	12 (11.7%)
Hispanic or Latino, n (%)	1 (2.5%)	1 (1.0%)
White, n (%)	35 (87.5%)	86 (83.5%)
Tobacco Use, n (%)	5 (12.5%)	10 (9.7%)
Comorbidities		
History of cancer	5 (12.5%)	5 (4.9%)
Coronary artery disease	1 (2.5%)	1 (1.0%)
Diabetes	2 (5.0%)	2 (1.9%)
Histologic Disease Activity		
Inactive, n (%)	-	34 (33.0%)
Active, n (%)		69 (67.0%)
Histology		
Normal, n (%)	-	21 (20.4%)
Quiescent, n (%)		13 (12.6%)
Mild, n (%)		31 (30.1%)
Moderate, n (%)		13 (12.6%)
Severe, n (%)		25 (24.3%)
Disease Location, n (%)		
Ileal, n (%)	-	7 (6.8%)
Colonic, n (%)		2 (1.9%)
Ileocolonic, n (%)		94 (91.3%)
Upper GI Involvement, n (%)		1 (1.0%)
Disease duration, years, median (25th − 75th percentile)	-	5 (2–9.5)
Perianal disease, n (%)	-	33 (32.0%)
Penetrating disease, n (%)	-	26 (25.2%)
Stricturing disease, n (%)	-	37 (35.9%)
Bowel Surgery, n (%)		
Ileocecal resection, n (%)		0 (0%)
SB resection, n (%)		0 (0%)
LB resection, n (%)		1 (1%)
Any IBD Therapy, n (%)	-	86 (83.5%)
Any 5-ASA, n (%)	-	14 (13.6%)
Steroid Therapy, n (%)	-	29 (28.2%)
Immunomodulator, n (%)	-	18 (17.5%)
Anti-TNFα Therapy, n (%)	-	40 (38.8%)
Vedolizumab, n (%)	-	10 (9.7%)
Ustekinumab, n (%)	-	20 (19.4%)
Patients with total SES available, n (%)	-	94 (91.3%)
Endoscopic Activity		
Inactive (SES 0–2), n (%)	-	28 (27.2%)
Active (SES ≥ 3), n (%)		66 (64.1%)
Not available, n (%)		9 (8.7%)
Clinical Activity		
Inactive (CDAI < 150), n (%)	-	62 (60.2%)
Active (CDAI ≥ 150), n (%)		41 (39.8%)
CRP, median (25th − 75th percentile)	-	6.35 (1.8–17.5)
Fecal calprotectin, median (25th − 75th percentile)	-	292.5 (144.3–886.3)

Age and BMI were compared using the Mann-Whitney U test. Categorical data was analyzed using the Pearson’s χ^2^ test, with Fisher’s exact test as appropriate (frequency ≤ 5). *p < 0.05, ***p < 0.001 vs. control. CRP (*n* = 98) and fecal calprotectin (*n* = 20) values for CD patients that were available within 6 months of colonoscopy were used in analysis.

## Serum analytes are increased in CD vs. Control

We sought to first determine cytokines and chemokines that were significantly altered in CD vs. controls. Of the 42 analytes assessed, 16 analytes were significantly increased in CD vs. controls with a false discovery rate of q < 0.05 (corresponding to a maximum p value of 0.016), including: TGFα, IFNα2, Fractalkine, PDGF-AA, IL13, IL25, MCP3, PDGF-ABBB, IL6, IL10, MCSF, GROα, IL4, IL17F, IL1β, and IL18 (Table 2).

**Table 2:** Serum cytokines and chemokines in control vs. CD.

Analyte	Control (*n* = 40)	CD (*n* = 103)	*p*	q
TGFα	3.29 (2.04–6.13)	6.29 (3.95–9.73)	**< 0.001**	**0.001**
IFNα2	4.06 (1.79–12.70)	12.67 (6.90–24.99)	**< 0.001**	**0.006**
Fractalkine	83.04 (52.60–112.32)	114.31 (76.63–187.15)	**0.001**	**0.018**
PDGF-AA	5878.00 (4542.50–7565.50)	7546.00 (5676.00–9620.50)	**0.002**	**0.018**
IL13	6.74 (0.73–32.49)	30.24 (8.84–68.35)	**0.002**	**0.020**
IL25	1153.00 (769.40–2210.50)	2182.50 (1286.25–3432.75)	**0.004**	**0.029**
IL6	0.88 (0.37–1.79)	2.16 (0.65–3.79)	**0.007**	**0.035**
MCP3	22.18 (14.14–33.32)	29.22 (21.58–39.88)	**0.006**	**0.035**
PDGF-ABBB	41872.00 (32268.00–51592.50)	49034.00 (36061.75–64495.50)	**0.007**	**0.035**
GROα	26.19 (19.53–39.49)	36.31 (24.29–55.42)	**0.012**	**0.039**
IL4	6.75 (5.52–8.35)	8.07 (6.38–9.88)	**0.012**	**0.039**
IL10	1.19 (0.52–2.37)	1.96 (0.82–5.62)	**0.011**	**0.039**
MCSF	9.61 (7.25–21.56)	23.25 (8.29–44.82)	**0.011**	**0.039**
IL1β	0.27 (0.21–1.71)	0.72 (0.27–8.91)	**0.015**	**0.041**
IL17F	5.27 (2.63–11.04)	13.2 (3.45–50.40)	**0.014**	**0.041**
IL18	73.15 (46.76–117.94)	94.39 (56.79–159.02)	**0.016**	**0.042**
FGF2	26.70 (18.44–58.52)	57.33 (28.07–100.69)	0.023	0.057
IP10	225.14 (170.01–265.66)	166.34 (125.04–252.25)	0.027	0.063
GCSF	54.83 (38.56–69.32)	60.83 (46.82–95.95)	0.030	0.064
IFNγ	1.64 (0.47–4.71)	2.87 (1.20–8.04)	0.031	0.064
IL1RA	3.08 (1.67–5.38)	4.40 (2.56–7.55)	0.032	0.064
IL12p70	0.74 (0.47–1.55)	1.12 (0.65–2.20)	0.038	0.072
MIP1β	41.13 (35.37–53.61)	37.84 (28.06–48.41)	0.055	0.101
IL2	0.08 (0.06–0.16)	0.16 (0.06–0.29)	0.065	0.105
IL9	14.77 (5.20–46.48)	30.70 (9.38–57.08)	0.065	0.105
MCP1	599.36 (520.86–742.30)	551.60 (414.59–682.39)	0.060	0.105
IL12p40	38.18 (19.57–54.08)	46.13 (21.10–107.16)	0.089	0.138
IL8	7.05 (5.57–8.99)	5.91 (4.37–8.87)	0.098	0.147
CXCL9	2594.00 (2051.00–3286.00)	2923.00 (2062.00–4750.50)	0.103	0.149
TNFα	22.80 (17.52–27.36)	27.56 (16.33–39.85)	0.109	0.153
FLT3L	14.43 (11.49–18.43)	13.08 (9.48–17.04)	0.134	0.181
VEGF-A	439.52 (195.92–534.62)	389.37 (270.05–670.21)	0.199	0.262
EGF	66.62 (45.94–104.81)	85.42 (51.67–127.86)	0.221	0.273
IL7	3.56 (2.16–5.33)	4.40 (2.59–6.73)	0.216	0.273
MDC	710.36 (593.52–841.51)	741.70 (557.35–1131.75)	0.350	0.419
MIP1α	8.16 (2.17–25.74)	11.21 (5.03–23.83)	0.369	0.430
sCD40L	9438.00 (5458.50 -16089.50)	8768.50 (5383.25–13017.25)	0.484	0.549
Eotaxin	117.91 (81.98–148.91)	122.03 (91.18–162.68)	0.539	0.581
IL5	2.67 (1.82–4.24)	3.08 (1.47–6.42)	0.535	0.581
IL15	2.78 (1.43–4.75)	2.72 (1.34–4.04)	0.740	0.777
IL1α	1.42 (1.31–2.76)	1.42 (1.23–4.58)	0.790	0.809
IL27	1512.00 (1201.50–1868.25)	1508.00 (1057.50–1953.75)	0.935	0.935

Data are presented as median (25th − 75th percentile) in pg/mL. p-values are calculated with a Mann Whitney U test and adjusted by the Benjamini-Hochberg (BH) method for multiple comparisons of all 42 analytes in order to calculate a false discovery rate, or q-value. 16 analytes met criteria for significance of q < 0.05. The following 5 cytokines were excluded from analysis because > 70% of the values were below the lower limit detection for the Luminex assay: GM-CSF, IL3, IL22, IL17A, TNFβ.

## Serum analytes are altered by disease activity

### Clinical disease activity

**Fig. 1:**
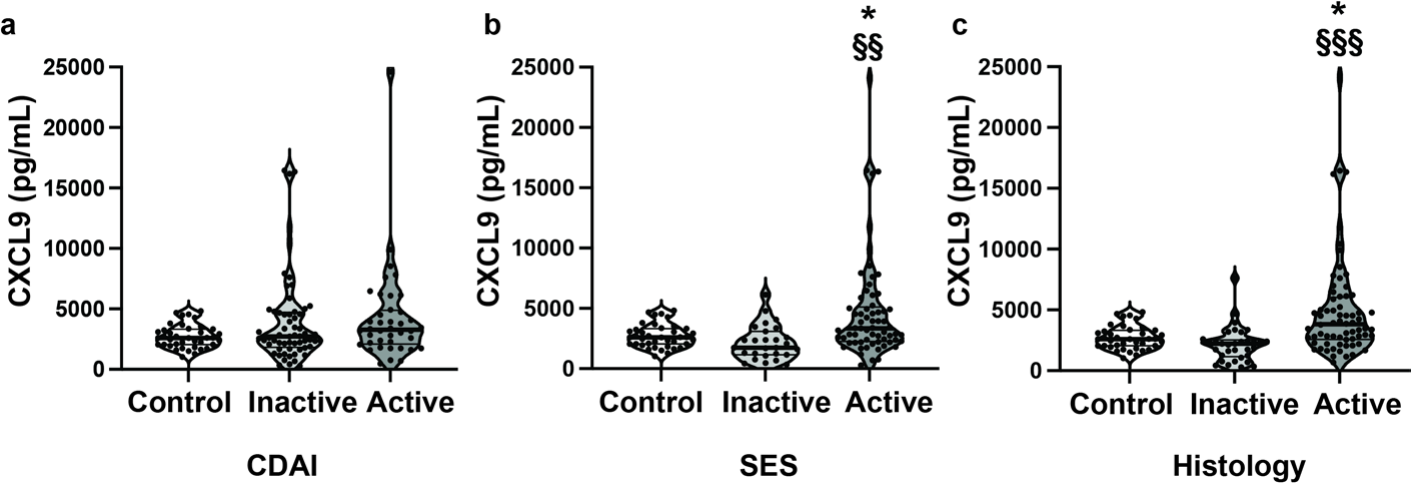
Serum CXCL9 is significantly increased in endoscopically and histologically, but not clinically, active vs. inactive CD. (**a**) Clinical disease activity by CDAI, (**b**) Endoscopic disease activity by SES, (**c**) Histologic disease activity. Bars represent median and quartiles. q-values were calculated by comparing disease activity (control vs. inactive vs. active) using Kruskal-Wallis test with post-hoc Dunn’s test, with BH adjustment for multiple comparisons of all 42 analytes, where q < 0.05 was considered significant. *q < 0.05 vs. control; ^§§^q < 0.01, ^§§§^q < 0.001 vs. inactive CD.

The Crohn’s Disease Activity Index (CDAI) was available for all 103 CD patients. 62 patients had clinically inactive disease and 41 had clinically active disease based on CDAI. There were 21 analytes that were significantly increased in inactive CD vs. controls: FGF2, Fractalkine, IFNα2, IFNγ, IL1β, IL1RA, IL2, IL4, IL9, IL10, IL12p70, IL13, IL25, IL17F, IL18, MCP3, MCSF, PDGF-AA, PDGF-ABBB, TGFα, and TNFα. Three analytes were significantly increased in clinically active CD vs. controls: IL6, PDGF-AA, and TGFα. There were no significantly altered cytokines and chemokines in comparison between clinically active vs. inactive CD (Table 3; Fig. 1a). The results of all 42 analytes assessed by disease activity are in Supplemental Table S2.

**Table 3:** Serum cytokines and chemokines are altered by clinical disease activity.

Analyte		Clinical (*n* = 103)	
	Control (*n* = 40)	Inactive (*n* = 62)	Active (*n* = 41)
FGF2	26.70 (18.44–58.52)	65.20 (31.94–100.83) *	42.14 (21.31–79.91)
Fractalkine	83.04 (52.60–112.32)	120.22 (77.08–189.56) **	104.82 (70.87–145.74)
IFNα2	4.06 (1.79–12.70)	13.68 (7.85–26.90) **	12.22 (4.88–19.38)
IFNγ	1.64 (0.47–4.71)	4.85 (1.35–10.66) *	2.17 (0.99–6.03)
IL1β	0.27 (0.21–1.71)	0.67 (0.27–10.18) *	0.72 (0.27–4.97)
IL1RA	3.08 (1.67–5.38)	4.71 (2.57–7.89) *	4.15 (2.39–6.66)
IL2	0.08 (0.06–0.16)	0.16 (0.08–0.30) *	0.08 (0.06–0.17)
IL4	6.75 (5.52–8.35)	8.23 (6.39–10.78) *	7.23 (5.75–9.47)
IL6	0.88 (0.37–1.79)	1.28 (0.43–3.34)	2.72 (1.36–4.39) *
IL9	14.77 (5.20–46.48)	37.54 (10.65–66.04) *	27.62 (7.89–39.58)
IL10	1.19 (0.52–2.37)	2.46 (0.79–6.15) *	1.67 (0.90–4.75)
IL12p70	0.74 (0.47–1.55)	1.24 (0.68–2.81) *	0.78 (0.60–1.65)
IL13	6.74 (0.73–32.49)	37.50 (11.92–81.76) **	14.04 (7.28–46.63)
IL25	1153.00 (769.40–2210.50)	2404.00 (1285.00–3621.00) *	2027.50 (1288.75–2452.25)
IL17F	5.27 (2.63–11.04)	13.94 (3.21–51.61) *	11.16 (3.46–36.78)
IL18	73.15 (46.76–117.94)	102.49 (76.45–163.51) *	84.04 (42.75–144.43)
MCP3	22.18 (14.14–33.32)	30.53 (22.65–45.03) *	23.25 (18.58–35.78)
MCSF	9.61 (7.25–21.56)	20.87 (9.80–45.03) *	25.11 (7.30–43.85)
PDGF-AA	5878.00 (4542.50–7565.50)	7159.00 (5712.00–9132.50) *	7955.00 (5683.00–10016.00) *
PDGF-ABBB	41872.00 (32268.00–51592.50)	52189.00 (38144.00–64284.00) *	47268.00 (35207.00–64556.00)
TGFα	3.29 (2.04–6.13)	6.39 (3.51–11.73) **	5.95 (4.42–8.40) *
TNFα	22.80 (17.52–27.36)	31.54 (21.13–43.26) *	20.33 (13.78–30.06)

Data are presented as median (25th − 75th percentile) in pg/mL. q-values were calculated by comparing clinical disease activity (control vs. inactive vs. active; inactive: CDAI < 150; active: CDAI ≥ 150), using the Kruskal-Wallis test with post-hoc Dunn’s test, with BH adjustment for multiple comparisons of all 42 analytes. Analytes that demonstrated significance in clinical activity assessment are shown in this table. For data for all 42 analytes, see Supplemental Table S2. q < 0.05 was considered significant. *q < 0.05, **q < 0.01 vs. control.

### Endoscopic disease activity

Of the 103 CD patients with serum samples, 94 had the total Simple Endoscopic Score for Crohn’s Disease (SES) score available. There were 28 CD patients with endoscopically inactive disease and 66 CD patients with endoscopically active disease. In comparison of endoscopically inactive CD vs. controls, there was a statistically significant increase in Fractalkine (145.97 [79.87–304.97] vs. 83.04 [52.60–112.32], q = 0.022). Nine analytes were significantly increased in endoscopically active CD vs. control including: TGFα, PDGF-AA, IFNα2, PDGF-AB/BB, GROα, Fractalkine, IL13, IL25, and CXCL9 (Table 4). MCP1 was significantly decreased in endoscopically active disease vs. control. There was no significant difference in either Fractalkine or MCP1 levels between endoscopically active vs. inactive CD. One chemokine, CXCL9, was significantly increased in endoscopically active CD vs. inactive CD (3348.00 [2290.00–5007.00] vs. 1743.00 [1204.00–2848.00], q = 0.001) (Fig. 1b).

**Table 4:** Serum cytokines and chemokines are altered by endoscopic disease activity.

Analyte		Endoscopy (*n* = 94)	
	Control (*n* = 40)	Inactive (*n* = 28)	Active (*n* = 66)
Fractalkine	83.04 (52.60–112.32)	145.97 (79.87–304.97) *	108.25 (78.03–174.43) *
GROα	26.19 (19.53–39.49)	29.87 (17.72–39.87)	40.64 (27.91–57.56) *
IFNα2	4.06 (1.79–12.70)	13.68 (7.53–20.42)	12.47 (5.44–25.52) **
IL13	6.74 (0.73–32.49)	41.98 (7.45–58.50)	28.96 (10.01–54.12) *
IL25	1153.00 (769.40–2210.50)	2309.00 (1492.75–3462.25)	2211.00 (1164.00–3444.00) *
MCP1	599.36 (520.86–742.30)	632.76 (476.48–783.20)	514.69 (411.60–638.74) *
CXCL9	2594.00 (2051.00–3286.00)	1743.00 (1204.00–2848.00)	3348.00 (2290.00–5007.00) *^§§^
PDGF-AA	5878.00 (4542.50–7565.50)	6314.00 (4790.50–7956.00)	8073.00 (5971.75–10126.50) **
PDGF-ABBB	41872.00 (32268.00–51592.50)	43823.00 (34509.50–56153.00)	54867.00 (39996.00–71631.00) **
TGFα	3.29 (2.04–6.13)	5.74 (3.36–9.56)	6.39 (4.46–9.62) **

Data are presented as median (25th − 75th percentile) in pg/mL. q-values were calculated by comparing endoscopic disease activity (control vs. inactive vs. active; inactive: SES 0–2; active: SES ≥ 3) using the Kruskal-Wallis test with post-hoc Dunn’s test, with BH adjustment for multiple comparisons of all 42 analytes. Analytes that demonstrated significance in endoscopic activity assessment are shown in this table. For data for all 42 analytes, see Supplemental Table S2. q < 0.05 was considered significant. *q < 0.05, **q < 0.01 vs. control; ^§§^q < 0.01 vs. inactive CD.

### Histologic disease activity

There was a statistically significant increase in serum TGFα (7.35 [3.91–11.73] vs. 3.29 [2.04–6.13], q = 0.023) and IFNα2 (13.18 [7.77–25.32] vs. 4.06 [1.79–12.70], q = 0.023) in histologically inactive CD vs. controls. A total of 17 analytes were significantly increased in histologically active CD vs. control, including: TGFα, PDGF-AA, Fractalkine, IL6, CXCL9, IFNα2, GROα, IL10, IL13, IFNγ, MCSF, GCSF, IL4, MCP3, PDGF-ABBB, IL1β, and IL25 (Table 5). There was no significant difference in either TGFα or IFNα2 levels between histologically active vs. inactive CD. One chemokine, CXCL9, was significantly increased in histologically active disease vs. inactive disease (3823.50 [2592.50–5934.00] vs. 2210.00 [1249.00–2447.00], q < 0.001) (Fig. 1c).

**Table 5:** Serum cytokines and chemokines are altered by histologic disease activity.

Analyte		Histology (*n* = 103)	
	Control (*n* = 40)	Inactive (*n* = 34)	Active (*n* = 69)
Fractalkine	83.04 (52.60–112.32)	95.36 (70.37–170.48)	119.79 (83.80–187.89) *
GCSF	54.83 (38.56–69.32)	56.13 (40.37–69.45)	63.12 (49.66–97.30) *
GROα	26.19 (19.53–39.49)	31.46 (17.35–44.47)	39.16 (26.56–60.93) *
IFNα2	4.06 (1.79–12.70)	13.18 (7.77–25.32) *	12.47 (5.24–23.48) *
IFNγ	1.64 (0.47–4.71)	1.67 (0.93–4.89)	4.83 (1.33–8.35) *
IL1β	0.27 (0.21–1.71)	0.67 (0.27–7.40)	0.85 (0.27–8.88) *
IL4	6.75 (5.52–8.35)	7.91 (6.39–9.27)	8.07 (6.36–10.25) *
IL6	0.88 (0.37–1.79)	1.03 (0.45–2.61)	2.59 (0.81–4.21) *
IL10	1.19 (0.52–2.37)	1.41 (0.42–5.30)	2.57 (1.07–5.74) *
IL13	6.74 (0.73–32.49)	21.25 (7.57–50.05)	32.17 (9.89–72.51) *
IL25	1153.00 (769.40–2210.50)	2560.00 (1310.00–3446.00)	2033.50 (1167.75–2924.00) *
MCP3	22.18 (14.14–33.32)	27.84 (18.50–42.97)	29.22 (21.85–38.96) *
MCSF	9.61 (7.25–21.56)	18.07 (7.66–37.90)	25.00 (10.23–51.28) *
CXCL9	2594.00 (2051.00–3286.00)	2210.00 (1249.00–2447.00)	3823.50 (2592.50–5934.00) *^§§§^
PDGF-AA	5878.00 (4542.50–7565.50)	6411.50 (5353.00–8232.50)	7955.00 (6053.00–9786.00) **
PDGF-ABBB	41872.00 (32268.00–51592.50)	51922.50 (35488.00–65252.00)	48724.00 (36979.75–64183.50) *
TGFα	3.29 (2.04–6.13)	7.35 (3.91–11.73) *	6.19 (4.03–9.45) **

Data are presented as median (25th − 75th percentile) in pg/mL. q-values were calculated by comparing histologic disease activity (control vs. inactive vs. active; inactive: normal and quiescent; active: mild, moderate, severe), using the Kruskal-Wallis test with post-hoc Dunn’s test, with BH adjustment for multiple comparisons of all 42 analytes. Analytes that demonstrated significance in histologic activity assessment are shown in this table. For data for all 42 analytes, see Supplemental Table S2. q < 0.05 was considered significant. *q < 0.05, **q < 0.01 vs. control; ^§§§^q < 0.001 vs. inactive CD.

### Effect of anti-TNFα therapy on serum inflammatory profile

In our CD cohort, 38.8% of patients were on anti-TNFα therapy. Thus, we sought to evaluate how current anti-TNFα therapy (*n* = 40) would affect serum inflammatory profiles. When patients on active anti-TNFα therapy were excluded from analysis, serum TNFα (32.42 [24.45–44.43] vs. 21.14 [15.77–28.24], q = 0.027) and IL5 (3.55 [1.95–9.88] vs. 1.61 [0.90–3.60], q = 0.032), in addition to CXCL9 (4579.00 [3096.00–6912.00] vs. 2285.50 [1729.25–2485.25], q < 0.001), were shown to be significantly increased in histologically active vs. inactive CD (Supplemental Table S3). This did not hold true in either clinical or endoscopic comparisons, where no analytes were found to be significantly different in either clinically or endoscopically active vs. inactive CD when current anti-TNFα therapy was considered. In clinically inactive disease vs. controls, there were several novel analytes that emerged as significantly elevated (IL4, PDGF-AA, PDGF-ABBB, and TNFα) in addition to TGFα and IFNα2 that were appreciated in the full cohort; however, this was not appreciated in endoscopic and histologic analyses.

### Only serum CXCL9 correlated with both endoscopic and histologic disease activity

**Fig. 2:**
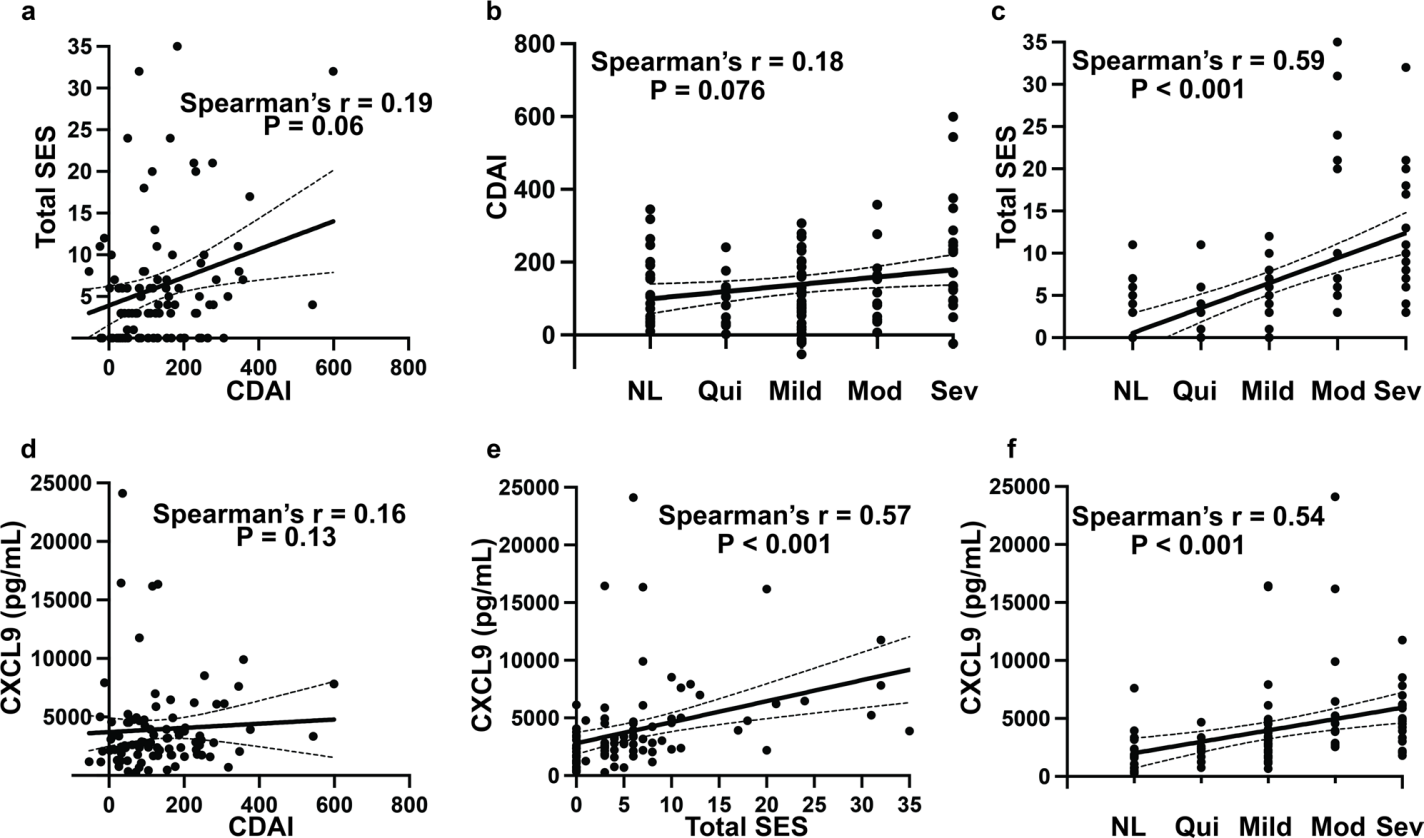
Correlation between disease activity indices and CXCL9. Using the cohort of CD patients for which all metrics of disease activity were available (*n* = 94), we assessed (**a**) CDAI vs. SES, (**b**) Histologic severity vs. CDAI, (**c**) Histologic severity vs. SES, (**d**) CDAI vs. CXCL9, (**e**) SES vs. CXCL9, and (**f**) Histologic severity vs. CXCL9 using Spearman’s correlation. NL = normal, Qui = quiescent, Mod = moderate, and Sev = severe. r = rho.

**Fig. 3:**
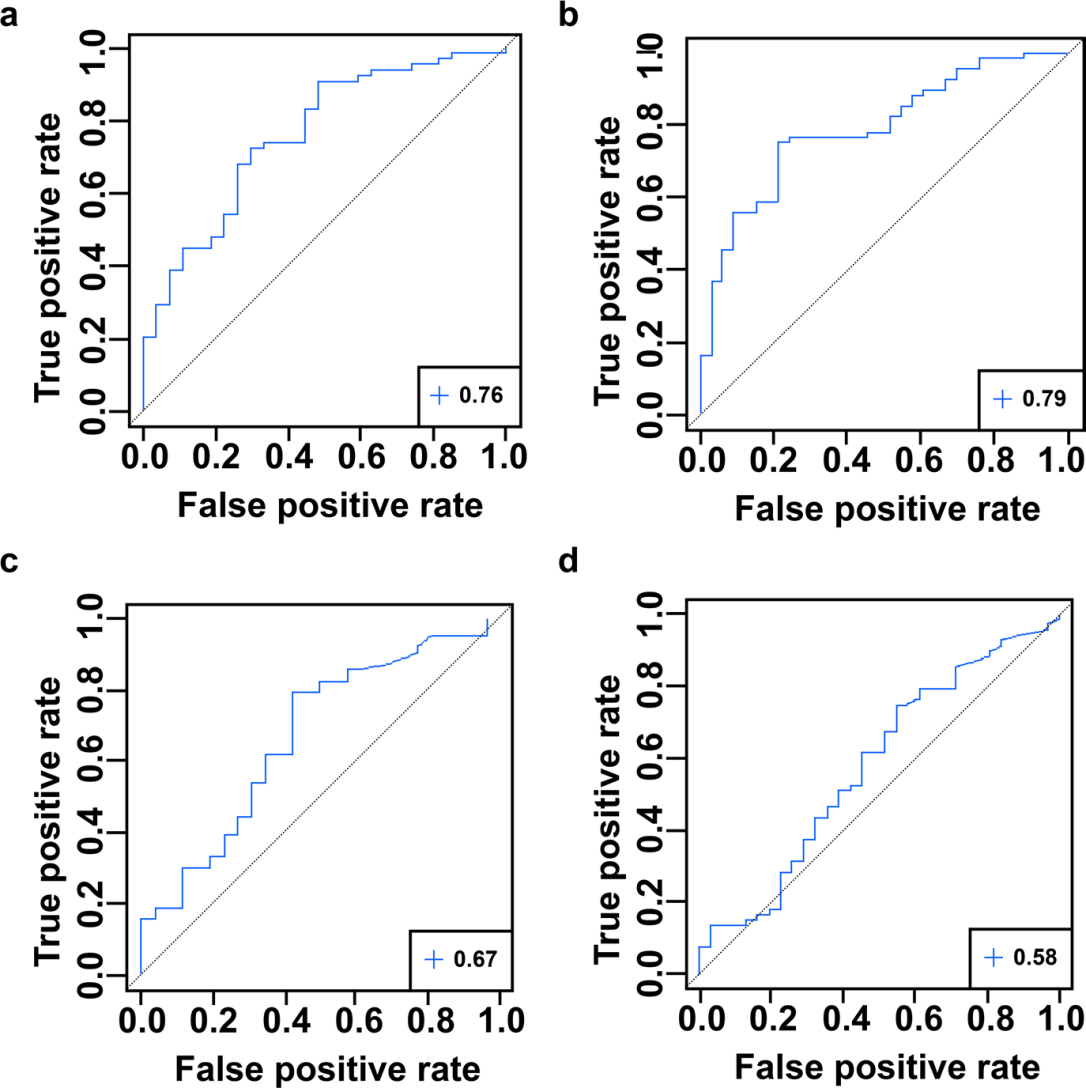
Receiver operating characteristic (ROC) curves with the area under the curve (AUC) demonstrating the discriminative capacity of serum CXCL9 and CRP to predict endoscopic and histologic disease activity (inactive vs. active). Shown are the AUC values of (**A**) CXCL9 for endoscopic activity is 0.76 (95% CI 0.65–0.87), (**B**) CXCL9 for histologic activity is 0.79 (95% CI 0.70–0.88), (**C**) CRP for endoscopic activity 0.67 (95% CI 0.54–0.79), and (**D**) CRP for histologic activity 0.58 (95% CI 0.45–0.70).

Clinical disease activity had a poor positive correlation with both endoscopic (*r* = 0.19, *p* = 0.060) and histologic (*r* = 0.18, *p* = 0.076) disease activity assessments (Fig. 2a and b, respectively). The total SES score had a significant positive correlation with histologic severity (normal, quiescent, mild, moderate, severe) (*r* = 0.59, *p* < 0.001) (Fig. 2c). After adjusting for multiple comparisons, the SES score was positively correlated to CXCL9 (*r* = 0.57, *p* < 0.001, q < 0.001) (Fig. 2e). After adjustment, histologic disease severity had a positive correlation to CXCL9 (*r* = 0.54, *p* < 0.001, q < 0.001) (Fig. 2f) and IL6 (*r* = 0.35, *p* = 0.001, q = 0.012) (Supplemental Table S4). There were no statistically significant correlations between serum analytes and clinical disease activity as measured by the CDAI (Fig. 2d, Supplemental Table S4). We generated a Receiver Operating Characteristic (ROC) curve using these analytes, as well as CRP, and calculated the area under the curve (AUC) to evaluate their ability to distinguish endoscopically and histologically inactive disease from active disease (Supplemental Table S5). Serum concentrations of CXCL9 had the highest discriminatory capacity for inactive vs. active CD both endoscopically (AUC = 0.76, 95% CI 0.65–0.87) and histologically (AUC = 0.79, 95% CI 0.70–0.88); in comparison to CRP, a currently established serum biomarker (Fig. 3). We were not able to fully assess the discriminative capacity of fecal calprotectin in our cohort as only 20 of the 103 CD patients (19%) had a fecal calprotectin available within 6 months of the study colonoscopy. Fecal calprotectin had a modest, but not significant, positive correlation with clinical (*r* = 0.35, *p* = 0.13), endoscopic (*r* = 0.38, *p* = 0.095), and histologic (*r* = 0.39, *p* = 0.081) disease activity assessments (Supplemental Fig. S1).

## Discussion

Currently, clinical and endoscopic remission are the treatment targets in CD. The role of histologic healing remains less clear, in part due to the paucity of studies relating histology to outcomes in CD. A recent meta-analysis identified a single study evaluating histologic activity and CD disease course, finding no significant association between histology and risk of relapse^14^. A study by Christensen et al., demonstrated improved clinical outcomes in ileal CD patients with histologic versus endoscopic healing. However, this study defined endoscopic healing purely as the absence of mucosal ulcerations, which does not factor in areas of stricturing or stenosis^15^. The classic patchy disease distribution of CD can impact the accuracy of both endoscopic and histologic disease assessments, and clinical disease assessments typically do not correlate well with active inflammation. Even the currently used biomarkers, serum CRP and fecal calprotectin, are not specific for active CD^5^. For example, elevated fecal calprotectin levels have been found to be associated with age > 65, obesity, medications including non-steroidal anti-inflammatory drugs and proton pump inhibitors, and irritable bowel syndrome^16^. The assessment of CRP and fecal calprotectin are useful, but other methodologies should be considered to identify additional non-invasive biomarker options that could aid in disease activity assessments and help to mitigate the cost of invasive procedures. In this study, we demonstrate that there are differences in the serum inflammatory profiles of CD patients vs. non-IBD controls and when CD patients are stratified by disease activity.

A number of analytes important in the inflammatory cascade and implicated in the pathogenesis of IBD were found to be altered in disease activity comparisons. Of note, many of the cytokines and chemokines that were significantly altered in endoscopically and histologically active CD vs. control, were similarly altered in clinically inactive CD vs. control. This should be interpreted in the context of poor correlation of clinical activity to both endoscopic and histologic disease activity, and in our cohort, > 60% of patients with clinically inactive disease by CDAI met criteria for either endoscopically and/or histologically active disease.

We sought to correlate serum analyte levels with measures of disease activity. In a study by Bourgonje et al., inflammatory biomarkers were correlated to SES, with serum amyloid A, IFN-γ, IL8, and IL17A showing a significantly positive correlation^7^. In our study, only CXCL9 had a strong significant positive correlation to both total SES and histologic severity, with a discriminative capacity of 0.76 and 0.79, respectively, and a better performance compared to CRP in distinguishing active from inactive CD. We were not able to fully incorporate fecal calprotectin into our analysis due to the low numbers of fecal calprotectin available in this cohort. Further studies evaluating CXCL9 in combination with other markers, at different thresholds, and in other inflammatory/non-IBD diseases are warranted, which would require a larger cohort. We also sought to evaluate the potential impact of biologic use on the inflammatory profile of our cohort. TNFα and IL5 were significantly increased in histologically active vs. inactive CD when patients on anti-TNFα therapy were excluded. This suggests that anti-TNFα therapies may help to modulate other cytokine signaling pathways besides TNFα. We could not assess the effect of other biologics (vedolizumab or ustekinumab) in this cohort given the smaller number of patients that were actively taking these medications.

CXCL9 is a chemokine which activates the chemokine receptor CXCR3 present on effector T cells and is important in T cell migration and leukocyte recruitment^17^. In CD, small bowel tissue contains resident memory T cells; a high concentration of pro-inflammatory markers in these cells, including IFNγ, could contribute to the production of chemokines (such as CXCL9), perpetuating chronic inflammation through continued immune cell migration^18,19^. In our study, CXCL9 was increased in active vs. inactive CD in endoscopic and histologic disease activity assessments, but not in clinical disease activity. In correlation analysis, it had a significant positive correlation to both histologic disease severity and total SES score, though the correlation to CDAI was not significant. CXCL9 has been shown to be related to disease activity in ulcerative colitis^20^. A small study by Kessel et al. examined serum inflammatory biomarkers predictive of relapse in IBD in 30 UC and 10 CD patients using Luminex technology, finding CXCL9 (as well as S100A8/A9, or serum calprotectin) to be a marker for unstable remission (i.e. associated with subsequent relapse), though it is unclear the contribution of IBD subtype had on these results as UC and CD were combined. This study used CDAI as their metric for disease remission vs. relapse, as histologic and endoscopic data was not available^21^. Boucher et al., studied serum analyte profiles based on disease location, noting that serum CXCL9 levels were more strongly associated with ileocolonic CD vs. either isolated ileal or colonic CD^6^. Given the dominance of ileocolonic disease in our cohort, it raises the question as to whether this significance is at least in part due to disease location. Our patient cohort only included 2 colonic only and 7 ileal only disease patients, so additional studies are needed to further assess CXCL9 as a potential biomarker in these subsets of CD patients. However, the fact that CXCL9 was only significantly increased in endoscopically or histologically active CD compared to inactive CD or controls supports its potential role as a biomarker of active disease.

While a strength of our study is our assessment of disease activity using three objective measures, two of which are validated scoring systems (SES and CDAI), in a well-characterized cohort of CD patients with a broad representation of CD phenotypes (perianal, structuring, and penetrating) and medication use, we recognize several limitations. This is a single center study, with a predominantly Caucasian cohort, and the majority of our CD patients were on active treatment including steroids, immunomodulators, and/or biologic therapies (Table 1), which could also modulate inflammatory profiles. The CD patients in our cohort predominantly had ileocolonic disease; we were thus unable to factor in disease location, either small bowel or colonic only disease, as a potential contributor to variations in inflammatory profiles. Our endoscopic disease assessment was determined at colonoscopy, which would not take into account more proximal small bowel disease if present. Additionally, there was a significant difference in age and BMI in our CD vs. control cohorts (though in both CD and control groups, the median BMI was in the overweight range). The age difference was due to our control group being recruited from patients undergoing routine colorectal cancer screening procedures, for which screening begins at age 45. However, there was no significant difference in age or BMI in the CD patients when categorized by inactive or active disease (Supplemental Table S1). There was also almost a complete absence of IBD-related surgeries in our cohort. Finally, our modality of choice, Luminex technology, is expensive and not readily accessible clinically, though narrowing biomarkers of interest down to a few assays would reduce the cost.

In conclusion, cytokine/chemokine profiling by Luminex technology can elicit differences between inflammatory profiles between CD and controls, and when stratified by disease activity. One chemokine, CXCL9, shows promise as a biomarker that differentiates between endoscopically and histologically active and inactive disease, though further studies evaluating its diagnostic predictive capacity with a more heterogenous cohort are certainly needed. Serum cytokine/chemokine profiling be a non-invasive strategy to reduce the need for frequent colonoscopy in CD to assess disease activity.

## Materials and methods

### Patients and study design

The study protocol was approved by the Institutional Review Board at Vanderbilt University Medical Center (VUMC). Written informed consent was obtained from all participants prior to sample collection and endoscopy, as part of the “Combinatorial Single Cell Strategies for a Crohn’s Disease Gut Cell Atlas”, identifier NCT04113733 (clinicaltrials.gov). Patients consented to the use of their demographic and clinical data as well as their serum and tissue samples as part of this study. Our group recently published a study focused on tissue single-cell RNA sequencing in a subset of this cohort^22^. All processes were in accordance with the guidelines and regulations of human subjects research.

Serum samples were prospectively obtained from CD patients or non-IBD controls at the time of colonoscopy. CD patients who were undergoing clinically indicated colonoscopy were recruited from the VUMC IBD Clinic from December 2019 to July 2023. The control cohort was composed of non-IBD patients undergoing colonoscopy at VUMC for colorectal cancer screening or polyp surveillance. Exclusion criteria included: <18 years of age, inability to provide consent, pregnancy, coagulopathy or bleeding disorder, renal or hepatic impairment, history of organ transplantation, and/or use of steroids, immunomodulators, or biologic therapy for a non-Crohn’s disease indication. After collection, study serum was processed within 2 h, snap frozen on dry ice, and then stored at − 80 °C. Demographic and clinical data were obtained for each subject at the time of serum collection and recorded in REDCap, including clinical disease activity as assessed by the Crohn’s Disease Activity Index (CDAI), current medication use, and disease distribution/characteristics for CD patients.

During colonoscopy, patients had clinical biopsies obtained in accordance with surveillance protocols for IBD or as needed for clinical indications. In addition, the CD and non-IBD controls had research biopsies obtained from the terminal ileum and ascending colon as part of the original research protocol, as the study was focused on ileocolonic disease.

### Assessment of disease severity

#### Clinical

Clinical disease severity was assessed using the CDAI, a validated patient-reported measuring tool used in clinical trials in which patients report the number of liquid stools, the extent of abdominal pain, general well-being, the occurrence of extraintestinal symptoms, the need for antidiarrheal drugs, the presence of abdominal masses, as well as more objective measures such as the hematocrit and body weight. The CDAI was calculated via a direct patient questionnaire about the prior 7 days at the time of colonoscopy and serum collection. Hematocrit and body weight were confirmed on chart review. The score ranges from 0 to 600, with < 150 corresponding to remission, or inactive disease, and ≥ 150 corresponding to active disease (mild, moderate, and severe)^23^.

#### Endoscopic

Endoscopic severity was determined by gastroenterologists specializing in IBD (D.B.B., R.L.D., B.S.P., E.A.S., S.N.H., D.A.S.) using the Simple Endoscopic Score for Crohn’s Disease (SES). The SES assesses four variables, each scored from 0 to 3, in each segment of the bowel (terminal ileum, ascending colon, transverse colon, descending colon, rectum), with a possible score ranging from 0 to 12 in each segment. These variables are the presence and size of ulcers, percentage of ulcerated surface, percentage of affected surface, and presence of stenosis^24^. The SES was recorded for the terminal ileum and ascending colon for all CD patients as per the original research protocol. The SES scores for the remaining segments of the colon were obtained from endoscopy reports. There were 9 patients for whom the SES scores of the remaining segments were not available, and they were eliminated from endoscopic disease activity assessment. Patients with a total SES score of 0–2 were defined as endoscopically inactive disease, whereas patients with SES of ≥ 3 were defined as endoscopically active disease^25^.

#### Histologic

Research and clinical biopsies in all patients were reviewed by a single collaborating gastrointestinal pathologist (M.K.W.), in a blinded manner. CD patient tissues were classified as: normal (normal tissue with no architectural distortion or inflammatory infiltrate), quiescent (architectural distortion, but no neutrophil infiltrate), mild (architectural distortion with mild increase in neutrophils in the lamina propria), moderate (architectural distortion with multiple clusters of neutrophils in the lamina propria or epithelium), or severe (significant architectural distortion/loss of crypts with granulation tissue or fibrinopurulent exudate) based on a modified Nancy Index^26,27^. Patients were considered to have histologically inactive CD if the most severe histologic classification from all biopsies obtained (either research or clinical) was normal or quiescent. Patients with mild, moderate, or severe pathology in any of their biopsies were considered to have histologically active CD.

### Serum cytokines and chemokines

Serum was obtained as above and analyzed using Milliplex™ MAP (Millipore, Billerica, MA) multiplex magnetic bead-based antibody detection kits according to manufacturer’s protocols on a FLEXMAP 3D® machine as previously described^13,27,28^. Luminex allows for multiple analytes to be assessed simultaneously in a small serum aliquot. Samples were analyzed with a pre-mixed 48 analyte kit on a 96 well plate format, with samples run in duplicate and designated wells for quality control. In total, 4 plates were assayed. The RANTES/CCL5 analyte was excluded given the requirement of a separate dilution protocol, which would have necessitated analysis on a separate plate. Five analytes that had more than 70% of values below the lower limit of detection were excluded (GM-CSF, IL3, IL17A, IL22, and TNFβ). Thus, 42 analytes remained in the final analysis.

### Statistical analysis

Analyte data are expressed as median (25th − 75th percentile) in pg/mL. Age, BMI, disease duration, CRP, and fecal calprotectin are expressed as median (25th − 75th percentile). Categorical variables are expressed as number (%), and analyzed using Pearson’s χ^2^ test, with Fisher’s exact test as appropriate. Outliers were removed using Grubb’s method^29^. Two group comparisons were performed with the Mann-Whitney U test. Comparisons of > 2 groups were performed by Kruskal-Wallis testing, with post-hoc pairwise comparison procedure with Dunn’s test. Correlations were determined using Spearman’s correlation coefficient. To account for multiple comparisons, a false discovery rate (q-value) was calculated using the Benjamini and Hochberg method adjustment^30^, with a q-value of < 0.05 considered significant. Receiver operating characteristic (ROC) curves were created for analytes that demonstrated a significant correlation in either endoscopic or histologic disease assessments to assess their ability to distinguish inactive vs. active disease. Statistical analysis was performed using R software (Version 4.2.2) and graphs were created using (GraphPad Prism 10).

## Supplementary Information

Below is the link to the electronic supplementary material.

Supplementary Material 1

Supplementary Material 2

## Data Availability

The datasets generated during and/or analyzed during the current study are available from the corresponding author on reasonable request.
