# Supplementary material for: Non-invasive determination of disease activity in Crohn’s disease by serum luminex profiling
Source: Sci Rep. 2026 Mar 9;16:8867. doi: 10.1038/s41598-026-42925-x (PMC12988101; doi:10.1038/s41598-026-42925-x)
Supplement: Supplementary file 1 — Supplementary Material 1 [file 41598_2026_42925_MOESM1_ESM.docx]

Supplemental Tables:

|  | **Clinical Activity (n=103)** | | | **Endoscopic Activity (n=94)** | | | **Histologic Activity (n=103)** | | |
| --- | --- | --- | --- | --- | --- | --- | --- | --- | --- |
|  | **Inactive**  **(n=62)** | **Active**  **(n=41)** | **p** | **Inactive**  **(n=28)** | **Active**  **(n=66)** | **p** | **Inactive**  **(n=34)** | **Active**  **(n=69)** | **p** |
| Age (median, 25^th^ - 75^th^) | 31.0 (23 - 47.8) | 32.0 (26 - 45) | 0.356 | 30 (25 - 43.5) | 31 (25 - 47.3) | 0.620 | 32.5 (25 - 45) | 31.0 (25 - 48) | 0.897 |
| Female Gender, n (%) | 35 (56.5%) | 28 (68.3%) | 0.317 | 19 (67.9%) | 40 (60.6%) | 0.666 | 24 (70.6%) | 39 (56.5%) | 0.245 |
| BMI (median, 25^th^ - 75^th^) | 26.0 (22.1 - 30.4) | 26.0 (21.7 - 29.9) | 0.666 | 25.9 (23.6 - 30.4) | 25.4 (21.1 - 30.2) | 0.365 | 25.4 (21 - 29.7) | 26.1 (22.3 - 30.3) | 0.512 |
| Race/Ethnicity, n (%)  Asian, n (%)  Black or AA, n (%)  Hispanic or Latino, n (%)  White, n (%) | 4 (6.5%)  6 (9.7%)  1 (1.6%)  51 (82.3%) | 0 (0%)  6 (14.6%)  0 (0%)  35 (85.4%) | 0.313 | 1 (3.6%)  3 (10.7%)  0 (0%)  24 (85.7%) | 2 (3.0%)  8 (12.1%)  1 (1.5%)  55 (83.3%) | 1 | 1 (2.9%)  3 (8.8%)  0 (0%)  30 (88.2%) | 3 (4.3%)  9 (13.0%)  1 (1.4%)  56 (81.2%) | 0.932 |
| Tobacco Use, n (%) | 4 (6.5%) | 6 (14.6%) | 0.191 | 4 (14.3%) | 6 (9.1%) | 0.478 | 4 (11.8%) | 6 (8.7%) | 0.726 |
| Histology  Normal, n (%)  Quiescent, n (%)  Mild, n (%)  Moderate, n (%)  Severe, n (%) | 11 (17.7%)  11 (17.7%)  21 (33.9%)  8 (12.9%)  11 (17.7%) | 10 (24.4%)  2 (4.9%)  10 (24.4%)  5 (12.2%)  14 (34.1%) | - | 12 (42.9%)  5 (17.9%)  11 (39.3%)  0 (0%)  0 (0%) | 8 (12.1%)  8 (12.1%)  19 (28.8%)  12 (18.2%)  19 (28.8%) | - | 21 (61.8%)  13 (38.2%)  0 (0%)  0 (0%)  0 (0%) | 0 (0%)  0 (0%)  31 (44.9%)  13 (18.8%)  25 (36.2%) | - |
| Ileocolonic Disease, n (%) | 59 (95.2%) | 35 (85.4%) | 0.151 | 23 (82.1%) | 62 (93.9%) | 0.120 | 28 (82.4%) | 66 (95.7%) | 0.0562 |
| Ileal Disease, n (%) | 2 (3.2%) | 5 (12.2%) | 0.112 | 4 (14.3%) | 3 (4.5%) | 0.191 | 6 (17.6%) | 1 (1.4%) | **0.005** |
| Colonic Disease, n (%) | 1 (1.6%) | 1 (2.4%) | 1 | 1 (3.6%) | 1 (1.5%) | 0.509 | 0 (0%) | 2 (2.9%) | 1 |
| Disease duration (median, 25^th^- 75^th^) | 5 (2 - 9.8) | 4 (2 - 9) | 0.661 | 5 (2 - 11.3) | 4 (2 - 8) | 0.622 | 5 (3 - 9.8) | 5 (2 - 9) | 0.885 |
| Perianal disease, n (%) | 16 (25.8%) | 17 (41.5%) | 0.147 | 5 (17.9%) | 24 (36.4%) | 0.091 | 9 (26.5%) | 24 (34.8%) | 0.532 |
| Penetrating disease, n (%) | 13 (21.0%) | 13 (31.7%) | 0.319 | 4 (14.3%) | 18 (27.3%) | 0.197 | 10 (29.4%) | 16 (23.2%) | 0.658 |
| Stricturing disease, n (%) | 21 (33.9%) | 16 (39.0%) | 0.746 | 6 (21.4%) | 27 (40.9%) | 0.116 | 15 (44.1%) | 22 (31.9%) | 0.318 |
| Any IBD Therapy, n (%) | 54 (87.1%) | 32 (78.0%) | 0.347 | 26 (92.9%) | 52 (78.8%) | 0.136 | 25 (73.5%) | 61 (88.4%) | 0.103 |
| Any 5-ASA, n (%) | 7 (11.3%) | 7 (17.1%) | 0.586 | 4 (14.3%) | 8 (12.1%) | 0.746 | 5 (14.7%) | 9 (13.0%) | 1 |
| Steroid Therapy, n (%) | 17 (27.4%) | 13 (31.7%) | 0.805 | 8 (28.6%) | 20 (30.3%) | 1 | 9 (26.5%) | 21 (30.4%) | 0.853 |
| Immunomodulator, n (%) | 5 (8.1%) | 13 (31.7%) | **0.003** | 3 (10.7%) | 13 (19.7%) | 0.377 | 4 (11.8%) | 14 (20.3%) | 0.410 |
| Anti-TNFα Therapy, n (%)  **+** Immunomodulator, n (%) | 27 (43.5%)  3 (4.8%) | 13 (31.7%)  5 (12.2%) | 0.317  0.260 | 12 (42.9%)  1 (3.6%) | 27 (40.9%)  6 (9.1%) | 1  0.67 | 13 (38.2%)  1 (2.9%) | 27 (39.1%)  7 (10.1%) | 1  0.266 |
| Vedolizumab, n (%) | 6 (9.7%) | 4 (9.8%) | 1 | 5 (17.9%) | 4 (6.1%) | 0.120 | 4 (11.8%) | 6 (8.7%) | 0.726 |
| Ustekinumab, n (%) | 11 (17.7%) | 9 (22.0%) | 0.784 | 6 (21.4%) | 12 (18.2%) | 0.937 | 4 (11.8%) | 16 (23.2%) | 0.196 |
| CDAI (median, 25^th^ - 75^th^) | 69 (27.3 - 99) | 235 (183 - 279) | - | 83.5 (35.5 - 193.5) | 124 (72.3 - 230) | - | 105 (45.3 - 163) | 118 (53 - 231) | - |
| CRP (median, 25^th^ - 75^th^) | 5.7 (1.5 – 15.9) | 8.4 (2.1 – 20.7) | - | 1.8 (0.8 – 11.7) | 9.3 (2.9 – 20.8) | - | 5.2 (1 – 16.1) | 8.2 (2.4 – 17.9) | - |
| Fecal calprotectin (median, 25^th^ - 75^th^) | 205 (134.5 - 365.3) | 662.5 (227.3 - 1275) | - | 189 (142 - 290) | 565 (192.5 - 1007.5) | - | 189 (142 - 591) | 295 (192.5 - 1265) | - |

**Supplemental Table S1.** Patient characteristics (Inactive vs. Active, by clinical, endoscopic, and histologic disease activity). Age, BMI, and disease duration were compared using the Mann-Whitney U test. Categorical data was analyzed using the Pearson's χ^2^ test, with Fisher’s exact test as appropriate (frequency ≤5).

| **Analyte** |  | **Clinical (n=103)** | | **Endoscopy (n=94)** | | **Histology (n=103)** | |
| --- | --- | --- | --- | --- | --- | --- | --- |
|  | **Control (n=40)** | **Inactive (n=62)** | **Active (n=41)** | **Inactive (n=28)** | **Active (n=66)** | **Inactive (n=34)** | **Active (n=69)** |
| sCD40L | 9438.00  (5458.50-16089.50) | 8302.00  (6011.00 - 11593.00) | 10768.50  (4560.50 - 13502.00) | 8453.00  (6614.75 - 11669.75) | 9143.00  (5356.00 - 13444.00) | 10709.00  (6547.00 – 13676.00) | 8472.00  (4643.00 - 11971.25) |
| EGF | 66.62  (45.94 - 104.81) | 87.95  (56.67 - 124.74) | 74.35  (40.96 - 122.51) | 78.60  (56.63 - 116.90) | 88.98  (61.53 - 135.48) | 75.74  (66.25 - 111.17) | 88.98  (42.31 - 128.90) |
| Eotaxin | 117.91  (81.98 - 148.91) | 128.07  (92.11 - 166.62) | 109.72  (88.81 - 152.19) | 115.16  (88.59 - 146.02) | 123.24  (92.45 - 163.46) | 121.13  (91.14 - 144.80) | 122.03  (91.21 - 168.41) |
| FGF2 | 26.70  (18.44 - 58.52) | 65.20 *  (31.94 - 100.83) | 42.14  (21.31 - 79.91) | 74.14  (43.05 - 116.57) | 42.95  (25.03 - 81.80) | 68.05  (31.15 - 108.65) | 43.93  (25.77 - 86.29) |
| FLT3L | 14.43  (11.49 - 18.43) | 13.79  (9.64 - 18.44) | 12.31  (9.28 - 16.17) | 13.16  (7.99 - 16.94) | 13.41  (9.64 - 17.02) | 12.08  (8.89 - 16.25) | 13.63  (9.98 - 17.05) |
| Fractalkine | 83.04  (52.60 - 112.32) | 120.22 **  (77.08 - 189.56) | 104.82  (70.87 - 145.74) | 145.97 *  (79.87 - 304.97) | 108.25 *  (78.03 - 174.43) | 95.36  (70.37 - 170.48) | 119.79 *  (83.80 - 187.89) |
| GCSF | 54.83  (38.56 - 69.32) | 58.21  (47.97 - 93.72) | 62.71  (44.31 - 96.62) | 57.63  (49.54 - 88.55) | 60.53  (46.04 - 99.35) | 56.13  (40.37 - 69.45) | 63.12 *  (49.66 - 97.30) |
| GROα | 26.19  (19.53 - 39.49) | 34.88  (23.94 - 54.14) | 39.41  (25.53 - 60.31) | 29.87  (17.72 - 39.87) | 40.64 *  (27.91 - 57.56) | 31.46  (17.35 - 44.47) | 39.16 *  (26.56 - 60.93) |
| IFNα2 | 4.06  (1.79 - 12.70) | 13.68 **  (7.85 - 26.90) | 12.22  (4.88 - 19.38) | 13.68  (7.53 - 20.42) | 12.47 **  (5.44 - 25.52) | 13.18 *  (7.77 - 25.32) | 12.47 *  (5.24 - 23.48) |
| IFNγ | 1.64  (0.47 - 4.71) | 4.85 *  (1.35 - 10.66) | 2.17  (0.99 - 6.03) | 2.24  (1.00 - 7.41) | 3.00  (1.19 - 8.14) | 1.67  (0.93 - 4.89) | 4.83 *  (1.33 - 8.35) |
| IL1α | 1.42  (1.31 - 2.76) | 1.42  (1.23 - 6.63) | 1.42  (0.97 - 2.00) | 1.42  (1.23 - 4.39) | 1.42  (1.23 - 4.56) | 1.41  (1.23 - 4.56) | 1.42  (1.23 - 4.20) |
| IL1β | 0.27  (0.21 - 1.71) | 0.67 *  (0.27 - 10.18) | 0.72  (0.27 - 4.97) | 1.36  (0.27 - 6.46) | 0.67  (0.27 - 8.92) | 0.67  (0.27 - 7.40) | 0.85 *  (0.27 - 8.88) |
| IL1RA | 3.08  (1.67 - 5.38) | 4.71 *  (2.57 - 7.89) | 4.15  (2.39 - 6.66) | 4.53  (2.41 - 6.34) | 4.36  (2.57 - 8.57) | 4.67  (2.99 - 7.00) | 4.25  (2.35 - 7.63) |
| IL2 | 0.08  (0.06 - 0.16) | 0.16 *  (0.08 - 0.30) | 0.08  (0.06 - 0.17) | 0.16  (0.08 - 0.35) | 0.11  (0.06 - 0.24) | 0.16  (0.08 - 0.39) | 0.10  (0.06 - 0.23) |
| IL4 | 6.75  (5.52 - 8.35) | 8.23 *  (6.39 - 10.78) | 7.23  (5.75 - 9.47) | 7.57  (6.20 - 10.03) | 8.07  (6.41 - 9.52) | 7.91  (6.39 - 9.27) | 8.07 *  (6.36 - 10.25) |
| IL5 | 2.67  (1.82 - 4.24) | 3.55  (1.62 - 6.03) | 2.46  (1.37 - 6.92) | 1.74  (1.17 - 7.47) | 3.20  (1.48 - 5.98) | 2.07  (1.11 - 5.00) | 3.29  (1.66 - 6.96) |
| IL6 | 0.88  (0.37 - 1.79) | 1.28  (0.43 - 3.34) | 2.72 *  (1.36 - 4.39) | 1.62  (0.57 - 2.60) | 2.14  (0.63 - 3.99) | 1.03  (0.45 - 2.61) | 2.59 *  (0.81 - 4.21) |
| IL7 | 3.56  (2.16 - 5.33) | 4.69  (3.17 - 6.59) | 4.03  (2.21 - 6.35) | 4.55  (3.60 - 7.13) | 3.89  (2.27 - 6.18) | 4.54  (2.86 - 6.76) | 3.89  (2.58 - 6.36) |
| IL8 | 7.05  (5.57 - 8.99) | 5.70  (4.61 - 8.95) | 5.91  (4.18 - 7.75) | 5.49  (4.10 - 6.82) | 5.70  (4.22 - 8.28) | 5.64  (4.45 - 7.30) | 6.16  (4.33 - 9.18) |
| IL9 | 14.77  (5.20 - 46.48) | 37.54 *  (10.65 - 66.04) | 27.62  (7.89 - 39.58) | 25.82  (6.35 - 41.71) | 34.35  (15.87 - 64.90) | 32.15  (8.48 - 42.31) | 30.70  (12.01 - 63.86) |
| IL10 | 1.19  (0.52 - 2.37) | 2.46 *  (0.79 - 6.15) | 1.67  (0.90 - 4.75) | 1.86  (0.55 - 5.10) | 2.09  (0.97 - 5.64) | 1.41  (0.42 - 5.30) | 2.57 *  (1.07 - 5.74) |
| IL12p40 | 38.18  (19.57 - 54.08) | 55.42  (25.77 - 106.87) | 33.16  (15.44 - 92.49) | 45.45  (23.44 - 91.66) | 49.87  (20.90 - 110.75) | 40.97  (15.01 - 82.50) | 48.34  (24.71 - 110.35) |
| IL12p70 | 0.74  (0.47 - 1.55) | 1.24 *  (0.68 - 2.81) | 0.78  (0.60 - 1.65) | 1.43  (0.67 - 2.05) | 0.81  (0.60 - 2.10) | 1.43  (0.68 - 2.81) | 0.90  (0.60 - 2.03) |
| IL13 | 6.74  (0.73 - 32.49) | 37.50 **  (11.92 - 81.76) | 14.04  (7.28 - 46.63) | 41.98  (7.45 - 58.50) | 28.96 *  (10.01 - 54.12) | 21.25  (7.57 - 50.05) | 32.17 *  (9.89 - 72.51) |
| IL15 | 2.78  (1.43 - 4.75) | 2.49  (1.22 - 4.09) | 2.97  (1.39 - 3.65) | 2.30  (1.21 - 3.59) | 2.88  (1.58 - 4.07) | 2.27  (1.19 - 3.77) | 2.89  (1.41 - 4.08) |
| IL25 | 1153.00  (769.40 - 2210.50) | 2404.00 *  (1285.00 - 3621.00) | 2027.50  (1288.75 - 2452.25) | 2309.00  (1492.75 - 3462.25) | 2211.00 *  (1164.00 - 3444.00) | 2560.00  (1310.00 - 3446.00) | 2033.50 *  (1167.75 - 2924.00) |

| **Analyte** |  | **Clinical (n=103)** | | **Endoscopy (n=94)** | | **Histology (n=103)** | |
| --- | --- | --- | --- | --- | --- | --- | --- |
|  | **Control (n=40)** | **Inactive (n=62)** | **Active (n=41)** | **Inactive (n=28)** | **Active (n=66)** | **Inactive (n=34)** | **Active (n=69)** |
| IL17F | 5.27  (2.63 - 11.04) | 13.94 *  (3.21 - 51.61) | 11.16  (3.46 - 36.78) | 13.35  (3.22 – 34.00) | 11.33  (3.47 - 46.77) | 11.51  (4.15 - 37.18) | 13.32  (2.50 - 52.92) |
| IL18 | 73.15  (46.76 - 117.94) | 102.49 *  (76.45 - 163.51) | 84.04  (42.75 - 144.43) | 107.32  (81.17 - 143.02) | 92.96  (53.60 - 169.76) | 93.97  (80.27 - 169.76) | 93.88  (53.55 - 156.26) |
| IL27 | 1512.00  (1201.50 - 1868.25) | 1510.00  (1091.00 - 1868.00) | 1497.00  (1023.75 - 1943.25) | 1303.00  (861.74 - 1629.50) | 1527.00  (1130.00 - 2058.00) | 1337.00  (904.46 - 1622.00) | 1583.00  (1063.00 - 2058.00) |
| IP10 | 225.14  (170.01 - 265.66) | 188.17  (122.98 - 289.49) | 155.14  (126.87 - 227.46) | 138.30  (108.57 - 217.40) | 169.40  (127.82 - 252.78) | 172.07  (106.67 - 207.25) | 160.75  (129.68 - 289.51) |
| MCP1 | 599.36  (520.86 - 742.30) | 556.93  (451.21 - 670.48) | 498.62  (411.60 - 686.36) | 632.76  (476.48 - 783.20) | 514.69 *  (411.60 - 638.74) | 591.83  (412.49 - 691.39) | 529.14  (427.57 - 670.00) |
| MCP3 | 22.18  (14.14 - 33.32) | 30.53 *  (22.65 - 45.03) | 23.25  (18.58 - 35.78) | 32.45  (20.82 - 44.00) | 27.91  (21.41 - 37.96) | 27.84  (18.50 - 42.97) | 29.22 *  (21.85 - 38.96) |
| MCSF | 9.61  (7.25 - 21.56) | 20.87 *  (9.80 - 45.03) | 25.11  (7.30 - 43.85) | 20.87  (8.83 - 35.86) | 24.75  (7.30 - 51.06) | 18.07  (7.66 - 37.90) | 25.00 *  (10.23 - 51.28) |
| MDC | 710.36  (593.52 - 841.51) | 709.91  (567.59 - 1080.50) | 825.64  (541.65 - 1251.00) | 729.47  (589.53 - 887.65) | 822.75  (566.51 - 1339.50) | 715.86  (552.46 - 884.05) | 758.37  (563.73 - 1181.00) |
| CXCL9 | 2594.00  (2051.00 – 3286.00) | 2714.00  (1884.00 - 4677.00) | 3276.00  (2157.25 - 4786.75) | 1743.00  (1204.00 - 2848.00) | 3348.00 *§§  (2290.00 - 5007.00) | 2210.00  (1249.00 - 2447.00) | 3823.50 * §§§  (2592.50 - 5934.00) |
| MIP1α | 8.16  (2.17 - 25.74) | 13.18  (4.82 - 32.35) | 9.67  (5.19 - 15.42) | 9.14  (4.63 - 20.12) | 13.18  (5.95 - 22.73) | 10.49  (4.82 - 18.99) | 12.02  (5.28 - 25.93) |
| MIP1β | 41.13  (35.37 - 53.61) | 39.89  (31.85 - 48.48) | 33.38  (24.99 - 48.21) | 40.97  (27.75 - 51.36) | 36.83  (28.59 - 47.17) | 40.36  (27.90 - 48.18) | 35.46  (28.24 - 48.57) |
| PDGF-AA | 5878.00  (4542.50 - 7565.50) | 7159.00 *  (5712.00 - 9132.50) | 7955.00 *  (5683.00 – 10016.00) | 6314.00  (4790.50 - 7956.00) | 8073.00 **  (5971.75 - 10126.50) | 6411.50  (5353.00 - 8232.50) | 7955.00 **  (6053.00 – 9786.00) |
| PDGF-ABBB | 41872.00  (32268.00 - 51592.50) | 52189.00 *  (38144.00 – 64284.00) | 47268.00  (35207.00 – 64556.00) | 43823.00  (34509.50 - 56153.00) | 54867.00 **  (39996.00 – 71631.00) | 51922.50  (35488.00 - 65252.00) | 48724.00 *  (36979.75 - 64183.50) |
| TGFα | 3.29  (2.04 - 6.13) | 6.39 **  (3.51 - 11.73) | 5.95 *  (4.42 - 8.40) | 5.74  (3.36 - 9.56) | 6.39 **  (4.46 - 9.62) | 7.35 *  (3.91 - 11.73) | 6.19 **  (4.03 - 9.45) |
| TNFα | 22.80  (17.52 - 27.36) | 31.54 *  (21.13 - 43.26) | 20.33  (13.78 - 30.06) | 27.07  (19.63 - 40.64) | 25.52  (15.16 - 34.87) | 21.66  (15.20 - 29.59) | 31.39  (19.16 - 41.00) |
| VEGFa | 439.52  (195.92 - 534.62) | 441.66  (300.29 - 675.93) | 355.80  (233.65 - 591.22) | 341.40  (210.50 - 460.67) | 391.25  (276.48 - 675.07) | 389.50  (157.49 - 675.93) | 389.24  (277.90 - 653.03) |

**Supplemental Table S2.** **Serum cytokines and chemokines are altered by disease activity (all 42 analytes).** Data are presented as median (25^th^ -75^th^ percentile) in pg/mL. q-values were calculated by comparing disease activity (control vs. inactive vs. active) for clinical (inactive: CDAI <150; Active: CDAI ≥150), endoscopic (inactive: SES 0-2; active: SES ≥3) and histologic measures (inactive: normal and quiescent; active: mild, moderate, severe), using the Kruskal-Wallis test with post-hoc Dunn’s test, with BH adjustment for multiple comparisons of all 42 analytes. q<0.05 was considered significant. q<0.05 was considered significant. *q<0.05, **q<0.01 vs. control; §§ q<0.01, §§§q<0.001 vs. inactive CD.

| **Analyte** |  | **Clinical (n=63)** | | **Endoscopy (n=55)** | | **Histology (n=63)** | |
| --- | --- | --- | --- | --- | --- | --- | --- |
|  | **Control (n=40)** | **Inactive (n=35)** | **Active (n=28)** | **Inactive (n=16)** | **Active (n=39)** | **Inactive (n=21)** | **Active (n=42)** |
| sCD40L | 9438.00  (5458.50-16089.50) | 9278.00  (6165.25 - 15165.50) | 11419.00  (4904.50 - 13560.00) | 10061.00  (7334.25 - 13823.75) | 10742.00  (5446.00 - 15914.25) | 11579.50  (7430.50 - 14400.00) | 9413.00  (4876.00 - 13251.00) |
| EGF | 66.62  (45.94 - 104.81) | 90.85  (55.37 - 123.02) | 71.29  (44.88 - 121.98) | 82.00  (59.78 - 109.01) | 88.47  (62.71 - 135.79) | 81.07  (69.90 - 122.74) | 87.95  (44.68 - 123.21) |
| Eotaxin | 117.91  (81.98 - 148.91) | 137.13  (107.36 - 191.56) | 101.57  (89.99 - 157.24) | 123.50  (93.18 - 182.49) | 134.07  (97.02 - 190.19) | 124.55  (93.92 - 168.45) | 132.96  (98.81 - 187.27) |
| FGF2 | 26.70  (18.44 - 58.52) | 59.50  (25.28 - 92.22) | 35.19  (17.59 - 70.09) | 65.20  (29.49 - 80.03) | 34.99  (22.05 - 68.99) | 59.50  (28.01 - 86.54) | 35.19  (20.09 - 74.14) |
| FLT3L | 14.43  (11.49 - 18.43) | 13.95  (10.53 - 18.74) | 13.51  (9.05 - 16.67) | 13.43  (8.28 - 16.94) | 14.16  (10.05 - 17.25) | 11.49  (8.75 - 14.70) | 14.34  (11.31 - 18.73) |
| Fractalkine | 83.04  (52.60 - 112.32) | 102.10  (74.68 - 181.48) | 95.36  (61.70 - 135.26) | 145.66  (86.18 - 186.49) | 95.36  (72.74 - 132.44) | 89.01  (68.47 - 147.00) | 102.30  (74.04 - 142.43) |
| GCSF | 54.83  (38.56 - 69.32) | 55.26  (46.09 - 73.48) | 66.53  (45.08 - 104.14) | 55.36  (47.46 - 63.36) | 61.92  (42.77 - 114.26) | 52.12  (40.37 - 63.03) | 61.92  (47.97 - 100.01) |
| GROα | 26.19  (19.53 - 39.49) | 36.31  (25.78 - 55.42) | 39.86  (27.59 - 64.75) | 33.59  (25.45 - 47.31) | 40.47 *  (29.59 - 65.11) | 36.10  (24.20 - 49.32) | 38.02 *  (26.68 - 62.79) |
| IFNα2 | 4.06  (1.79 - 12.70) | 11.63 *  (5.81 - 25.10) | 12.22  (4.39 - 19.65) | 12.22  (7.53 - 19.65) | 11.98 *  (5.33 - 24.27) | 12.22  (7.77 - 25.17) | 11.77  (4.61 - 22.17) |
| IFNγ | 1.64  (0.47 - 4.71) | 3.94  (1.37 - 7.37) | 2.09  (0.98 - 3.91) | 2.09  (1.16 - 3.81) | 2.73  (1.09 - 6.62) | 1.61  (0.86 - 3.07) | 2.88  (1.36 - 7.28) |
| IL1α | 1.42  (1.31 - 2.76) | 1.42  (1.39 - 7.03) | 1.42  (1.10 - 2.11) | 1.54  (1.41 - 7.31) | 1.42  (1.23 - 2.42) | 1.42  (1.23 - 2.02) | 1.42  (1.23 - 5.94) |
| IL1β | 0.27  (0.21 - 1.71) | 0.67  (0.21 - 9.86) | 0.67  (0.27 - 2.26) | 1.36  (0.24 - 5.78) | 0.67  (0.23 - 2.19) | 0.67  (0.26 - 2.45) | 0.67  (0.21 - 4.63) |
| IL1RA | 3.08  (1.67 - 5.38) | 4.81  (3.00 - 7.55) | 4.20  (2.89 - 6.51) | 4.53  (2.78 - 6.29) | 4.35  (3.08 - 7.35) | 4.76  (2.99 - 6.85) | 4.15  (2.57 - 7.39) |
| IL2 | 0.08  (0.06 - 0.16) | 0.10  (0.06 - 0.17) | 0.08  (0.06 - 0.16) | 0.12  (0.08 - 0.18) | 0.08  (0.06 - 0.16) | 0.16  (0.08 - 0.20) | 0.08  (0.06 - 0.16) |
| IL4 | 6.75  (5.52 - 8.35) | 8.31 *  (7.01 - 10.39) | 7.20  (6.08 - 8.89) | 7.31  (6.71 - 8.28) | 8.18  (6.80 - 9.82) | 7.18  (6.47 - 8.43) | 8.23 *  (6.92 - 10.50) |
| IL5 | 2.67  (1.82 - 4.24) | 3.72  (1.64 - 7.32) | 2.35  (1.48 - 6.63) | 1.48  (0.60 - 2.98) | 3.29  (1.64 - 6.27) | 1.61  (0.90 - 3.60) | 3.55 §  (1.95 - 9.88) |
| IL6 | 0.88  (0.37 - 1.79) | 1.77  (0.74 - 3.68) | 2.84 *  (1.90 - 5.24) | 2.54  (1.15 - 3.13) | 2.42 **  (0.97 - 4.61) | 1.03  (0.66 - 3.12) | 2.84 ***  (1.61 - 6.27) |
| IL7 | 3.56  (2.16 - 5.33) | 4.53  (3.35 - 7.51) | 4.55  (2.61 - 7.06) | 4.82  (3.76 - 7.83) | 4.32  (2.60 - 7.46) | 4.63  (3.74 - 7.12) | 3.90  (2.82 - 7.34) |
| IL8 | 7.05  (5.57 - 8.99) | 6.18  (4.68 - 9.59) | 6.28  (4.24 - 10.13) | 5.01  (4.33 - 7.71) | 6.01  (4.42 - 9.13) | 5.21  (4.40 - 7.26) | 7.58  (4.85 - 9.83) |
| IL9 | 14.77  (5.20 - 46.48) | 29.37  (7.57 - 57.08) | 27.70  (11.17 - 40.33) | 24.01  (8.71 - 37.04) | 30.70  (12.38 - 57.08) | 29.14  (10.20 - 39.40) | 27.85  (7.39 - 63.35) |
| IL10 | 1.19  (0.52 - 2.37) | 3.29  (0.78 - 5.56) | 1.65  (1.09 - 4.09) | 1.86  (0.70 - 5.10) | 2.29  (0.83 - 4.57) | 1.31  (0.74 - 5.37) | 2.61 *  (1.19 - 4.83) |
| IL12p40 | 38.18  (19.57 - 54.08) | 61.03  (29.37 - 111.46) | 33.94  (15.10 - 145.91) | 62.75  (36.28 - 116.69) | 52.65  (21.90 - 153.98) | 51.79  (10.59 - 111.06) | 55.42  (27.11 - 164.48) |
| IL12p70 | 0.74  (0.47 - 1.55) | 1.09  (0.70 - 2.75) | 0.74  (0.60 - 1.56) | 1.55  (0.88 - 1.96) | 0.76  (0.60 - 1.72) | 1.34  (0.73 - 2.18) | 0.78  (0.60 - 1.77) |
| IL13 | 6.74  (0.73 - 32.49) | 27.78  (10.02 - 51.56) | 12.41  (7.01 - 46.02) | 21.25  (6.62 - 49.59) | 16.03  (8.06 - 44.66) | 16.03  (7.24 - 42.02) | 23.48  (8.60 - 75.85) |
| IL15 | 2.78  (1.43 - 4.75) | 2.29  (1.20 - 3.40) | 3.02  (2.30 - 3.57) | 2.29  (1.06 - 3.49) | 2.77  (2.12 - 3.71) | 2.27  (1.19 - 3.02) | 2.95  (1.42 - 4.09) |
| IL25 | 1153.00  (769.40 - 2210.50) | 1649.00  (1071.50 - 3498.75) | 1836.00  (1221.50 - 2417.00) | 2115.00  (1545.25 - 2983.00) | 1793.50  (1031.75 - 2891.25) | 2191.00  (1310.00 - 3444.00) | 1620.00  (1047.00 - 2793.00) |

| **Analyte** |  | **Clinical (n=63)** | | **Endoscopy (n=55)** | | **Histology (n=63)** | |
| --- | --- | --- | --- | --- | --- | --- | --- |
|  | **Control (n=40)** | **Inactive (n=35)** | **Active (n=28)** | **Inactive (n=16)** | **Inactive (n=39)** | **Active (n=21)** | **Inactive (n=42)** |
| IL17F | 5.27  (2.63 - 11.04) | 11.42  (2.72 - 50.40) | 13.29  (2.81 - 34.59) | 11.51  (2.55 - 28.47) | 8.69  (2.68 - 36.32) | 8.76  (3.58 - 28.49) | 14.69  (2.45 - 59.36) |
| IL18 | 73.15  (46.76 - 117.94) | 91.88  (68.61 - 169.51) | 85.54  (46.16 - 148.86) | 104.58  (74.64 - 148.96) | 90.41  (56.55 - 208.67) | 91.55  (77.95 - 163.51) | 92.20  (53.40 - 159.45) |
| IL27 | 1512.00  (1201.50 - 1868.25) | 1475.00  (1197.50 - 1746.50) | 1506.00  (1042.00 - 1948.50) | 1253.00  (861.74 - 1780.00) | 1497.00  (1197.50 - 1836.50) | 1350.50  (883.10 - 1701.00) | 1518.50  (1244.00 - 1991.50) |
| IP10 | 225.14  (170.01 - 265.66) | 226.21  (158.16 - 320.99) | 175.38  (139.24 - 237.01) | 172.07  (134.82 - 241.29) | 214.94  (155.65 - 281.72) | 204.86  (160.08 - 233.84) | 224.27  (145.99 - 301.07) |
| MCP1 | 599.36  (520.86 - 742.30) | 595.34  (489.95 - 731.33) | 575.28  (427.62 - 735.02) | 670.48  (571.22 - 788.06) | 551.64  (449.66 - 699.47) | 598.39  (466.68 - 716.00) | 556.13  (449.14 - 747.84) |
| MCP3 | 22.18  (14.14 - 33.32) | 27.88  (23.05 - 38.50) | 22.65  (18.56 - 35.12) | 28.07  (22.60 - 35.12) | 25.76  (18.88 - 35.76) | 27.57  (18.94 - 35.29) | 27.91  (21.12 - 37.96) |
| MCSF | 9.61  (7.25 - 21.56) | 21.00  (10.49 - 43.88) | 26.39  (9.24 - 45.07) | 20.58  (9.56 - 32.54) | 25.82  (10.49 - 49.87) | 19.33  (7.56 - 32.25) | 25.25 *  (10.81 - 51.06) |
| MDC | 710.36  (593.52 - 841.51) | 695.18  (608.93 - 993.15) | 729.47  (534.85 - 1173.00) | 705.52  (601.71 - 800.14) | 830.95  (608.21 - 1134.50) | 729.47  (620.93 - 1037.00) | 695.18  (553.70 - 1128.00) |
| CXCL9 | 2594.00  (2051.00 – 3286.00) | 3023.50  (2368.75 - 4952.25) | 3789.00  (2490.00 - 6118.50) | 2380.00  (1720.50 - 3624.00) | 3619.50 **  (2513.75 - 6377.25) | 2285.50  (1729.25 - 2485.25) | 4579.00 ***§§§  (3096.00 – 6912.00) |
| MIP1α | 8.16  (2.17 - 25.74) | 10.30  (4.96 - 24.53) | 8.90  (5.11 - 14.59) | 9.14  (5.33 - 15.37) | 10.66  (3.48 - 18.86) | 10.49  (6.07 - 15.89) | 8.65  (4.46 - 18.45) |
| MIP1β | 41.13  (35.37 - 53.61) | 45.85  (38.98 - 61.07) | 35.10  (27.57 - 44.50) | 43.73  (35.15 - 52.88) | 40.74  (33.59 - 51.14) | 41.13  (36.85 - 51.36) | 39.89  (31.55 - 52.03) |
| PDGF-AA | 5878.00  (4542.50 - 7565.50) | 7546.00 *  (6067.50 - 9638.50) | 8216.00  (5631.00 - 10142.75) | 6058.00  (4985.00 - 7588.50) | 8643.00 **  (6444.50 - 10462.50) | 6397.00  (5446.00 - 8242.00) | 8368.00 **  (6557.00 - 10008.25) |
| PDGF-ABBB | 41872.00  (32268.00 - 51592.50) | 55871.50 *  (39829.00 - 68527.75) | 51041.00  (34993.00 - 72381.50) | 40864.00  (32839.50 - 52498.50) | 64556.00 **  (48354.50 - 79248.00) | 56151.00  (35370.00 - 69125.00) | 53314.50 *  (37798.50 - 73882.50) |
| TGFα | 3.29  (2.04 - 6.13) | 6.04 *  (3.51 - 11.20) | 5.74  (4.72 - 7.50) | 5.38  (3.68 - 9.18) | 6.38 **  (5.06 - 8.73) | 5.64  (4.02 - 9.10) | 5.99 **  (4.13 - 8.19) |
| TNFα | 22.80  (17.52 - 27.36) | 32.37 *  (22.78 - 42.11) | 26.66  (16.64 - 32.32) | 26.66  (20.74 - 33.51) | 29.46  (18.40 - 37.18) | 21.14  (15.77 - 28.24) | 32.42 ** §  (24.45 - 44.43) |
| VEGFa | 439.52  (195.92 - 534.62) | 386.42  (301.43 - 638.86) | 336.83  (215.53 - 540.79) | 341.40  (210.50 - 454.82) | 383.59  (251.23 - 625.20) | 325.96  (149.56 - 488.31) | 390.82  (288.34 - 717.89) |

**Supplemental Table S3.** **Serum cytokines and chemokines are altered by disease activity when patients who use anti-TNFα are excluded (all 42 analytes).** Data are presented as median (25^th^ -75^th^ percentile) in pg/mL. We performed cytokine/chemokine analysis by disease activity only including patients who were not on anti-TNF therapy at the time of blood draw. q-values were calculated by comparing disease activity (control vs. inactive vs. active) for clinical (inactive: CDAI <150; Active: CDAI ≥150), endoscopic (inactive: SES 0-2; active: SES ≥3) and histologic measures (inactive: normal and quiescent; active: mild, moderate, severe), using the Kruskal-Wallis test with post-hoc Dunn’s test, with BH adjustment for multiple comparisons of all 42 analytes. q<0.05 was considered significant. q<0.05 was considered significant. *q<0.05, **q<0.01, ***q<0.001 vs. control; §q<0.05, §§§q<0.001 vs. inactive CD.

| **Analyte** | **Analyte vs. CDAI** | | | **Analyte vs. SES** | | | **Analyte vs. Histology** | | |
| --- | --- | --- | --- | --- | --- | --- | --- | --- | --- |
|  | **rho** | **p** | **q** | **rho** | **p** | **q** | **rho** | **p** | **q** |
| sCD40L | 0.11 | 0.307 | 0.729 | -0.15 | 0.147 | 0.418 | -0.14 | 0.168 | 0.442 |
| EGF | -0.06 | 0.585 | 0.847 | -0.15 | 0.149 | 0.418 | -0.09 | 0.390 | 0.636 |
| Eotaxin | 0.01 | 0.926 | 0.966 | -0.04 | 0.715 | 0.790 | -0.02 | 0.881 | 0.925 |
| FGF2 | -0.17 | 0.107 | 0.728 | -0.22 | 0.034 | 0.218 | -0.21 | 0.041 | 0.286 |
| FLT3L | -0.07 | 0.479 | 0.774 | -0.06 | 0.566 | 0.706 | 0.08 | 0.419 | 0.636 |
| Fractalkine | -0.09 | 0.388 | 0.729 | -0.12 | 0.255 | 0.502 | 0.07 | 0.514 | 0.677 |
| GCSF | 0.02 | 0.874 | 0.941 | 0.19 | 0.070 | 0.327 | 0.20 | 0.055 | 0.286 |
| GROα | 0.13 | 0.207 | 0.729 | 0.22 | 0.036 | 0.218 | 0.14 | 0.196 | 0.477 |
| IFNα2 | -0.10 | 0.331 | 0.729 | -0.06 | 0.556 | 0.706 | -0.12 | 0.265 | 0.531 |
| IFNγ | -0.03 | 0.802 | 0.914 | 0.06 | 0.574 | 0.706 | 0.17 | 0.112 | 0.337 |
| IL1α | -0.11 | 0.306 | 0.729 | -0.15 | 0.144 | 0.418 | -0.06 | 0.596 | 0.732 |
| IL1β | -0.01 | 0.955 | 0.966 | -0.01 | 0.929 | 0.929 | 0.08 | 0.439 | 0.636 |
| IL1RA | -0.08 | 0.468 | 0.774 | 0.10 | 0.349 | 0.564 | -0.08 | 0.457 | 0.639 |
| IL2 | -0.16 | 0.119 | 0.728 | -0.16 | 0.123 | 0.418 | -0.18 | 0.088 | 0.291 |
| IL4 | -0.03 | 0.792 | 0.914 | -0.01 | 0.901 | 0.924 | 0.08 | 0.428 | 0.636 |
| IL5 | -0.06 | 0.574 | 0.847 | 0.06 | 0.541 | 0.706 | 0.11 | 0.305 | 0.583 |
| IL6 | 0.32 | 0.002 | 0.086 | 0.27 | 0.009 | 0.122 | 0.35 | 0.001 | **0.012** |
| IL7 | -0.04 | 0.727 | 0.914 | -0.10 | 0.348 | 0.564 | -0.07 | 0.516 | 0.677 |
| IL8 | 0.04 | 0.717 | 0.914 | 0.13 | 0.226 | 0.484 | 0.12 | 0.260 | 0.531 |
| IL9 | -0.19 | 0.064 | 0.728 | 0.04 | 0.694 | 0.788 | 0.01 | 0.928 | 0.938 |
| IL10 | -0.07 | 0.520 | 0.808 | 0.08 | 0.446 | 0.669 | 0.16 | 0.128 | 0.357 |
| IL12p40 | 0.00 | 0.966 | 0.966 | 0.06 | 0.589 | 0.706 | 0.10 | 0.361 | 0.636 |
| IL12p70 | -0.11 | 0.286 | 0.729 | -0.12 | 0.263 | 0.502 | -0.13 | 0.205 | 0.477 |
| IL13 | -0.09 | 0.393 | 0.729 | -0.09 | 0.398 | 0.619 | 0.01 | 0.938 | 0.938 |
| IL15 | 0.09 | 0.367 | 0.729 | 0.14 | 0.168 | 0.441 | 0.21 | 0.048 | 0.286 |
| IL25 | -0.15 | 0.139 | 0.728 | -0.13 | 0.225 | 0.484 | -0.18 | 0.082 | 0.291 |
| IL17F | -0.09 | 0.384 | 0.729 | -0.11 | 0.306 | 0.535 | -0.18 | 0.088 | 0.291 |
| IL18 | -0.16 | 0.126 | 0.728 | -0.11 | 0.282 | 0.515 | -0.21 | 0.044 | 0.286 |
| IL27 | 0.11 | 0.290 | 0.729 | 0.24 | 0.022 | 0.218 | 0.22 | 0.031 | 0.286 |
| IP10 | -0.08 | 0.422 | 0.738 | 0.13 | 0.203 | 0.484 | 0.02 | 0.827 | 0.891 |
| MCP1 | -0.09 | 0.399 | 0.729 | -0.19 | 0.062 | 0.326 | -0.05 | 0.645 | 0.732 |
| MCP3 | -0.13 | 0.206 | 0.729 | -0.07 | 0.526 | 0.706 | -0.05 | 0.625 | 0.732 |
| MCSF | -0.02 | 0.843 | 0.932 | 0.05 | 0.656 | 0.765 | 0.09 | 0.392 | 0.636 |
| MDC | 0.03 | 0.796 | 0.914 | 0.23 | 0.028 | 0.218 | 0.19 | 0.068 | 0.291 |
| CXCL9 | 0.16 | 0.132 | 0.728 | 0.57 | <0.001 | **<0.001** | 0.54 | <0.001 | **<0.001** |
| MIP1α | -0.11 | 0.284 | 0.729 | -0.06 | 0.584 | 0.706 | -0.03 | 0.798 | 0.882 |
| MIP1β | -0.17 | 0.103 | 0.728 | -0.16 | 0.135 | 0.418 | -0.05 | 0.632 | 0.732 |
| PDGF-AA | 0.15 | 0.160 | 0.729 | 0.31 | 0.002 | **0.044** | 0.30 | 0.004 | 0.053 |
| PDGF-ABBB | 0.04 | 0.723 | 0.914 | 0.16 | 0.127 | 0.418 | 0.08 | 0.418 | 0.636 |
| TGFα | -0.03 | 0.805 | 0.914 | -0.01 | 0.902 | 0.924 | -0.06 | 0.580 | 0.732 |
| TNFα | -0.11 | 0.277 | 0.729 | -0.02 | 0.862 | 0.924 | 0.13 | 0.229 | 0.507 |
| VEGFa | 0.04 | 0.708 | 0.914 | 0.13 | 0.230 | 0.484 | 0.18 | 0.090 | 0.291 |
| CRP | 0.16 | 0.13 | - | 0.39 | **<0.001** | - | 0.25 | **0.018** | - |

**Supplemental Table S4.** **Serum cytokine and chemokine correlate to disease activity.** In this analysis, we used the cohort of patients for which all metrics of disease activity were available (n=94). Spearman’s correlation was used to compare analytes vs. disease activity indices. q-values of analytes that demonstrated significance in correlation analysis after adjusting for multiple comparisons are shown underlined and bolded. Spearman’s correlation of CRP to activity assessments is included with corresponding rho and p-values.

|  | **AUC for endoscopic activity** | **AUC for histologic activity** |
| --- | --- | --- |
|  |  |  |
| **CXCL9** | 0.76 (0.65-0.87) | 0.79 (0.70-0.88) |
| **PDGF-AA** | 0.66 (0.55-0.78) | 0.61 (0.49-0.73) |
| **IL6** | 0.57 (0.45-0.69) | 0.64 (0.53-0.75) |
| **CRP** | 0.67 (0.54-0.79) | 0.58 (0.45-0.70) |

**Supplemental Table S5**. Area under the curve (AUC) for analytes that yielded a significantly positive correlation to either total SES or histologic severity. Values in parentheses are 95% confidence interval.
