# Supplementary material for: Non-invasive determination of disease activity in Crohn’s disease by serum luminex profiling
Source: Sci Rep. 2026 Mar 9;16:8867. doi: 10.1038/s41598-026-42925-x (PMC12988101; doi:10.1038/s41598-026-42925-x)
Supplement: Supplementary file 2 — Supplementary Material 2 [file 41598_2026_42925_MOESM2_ESM.pdf]

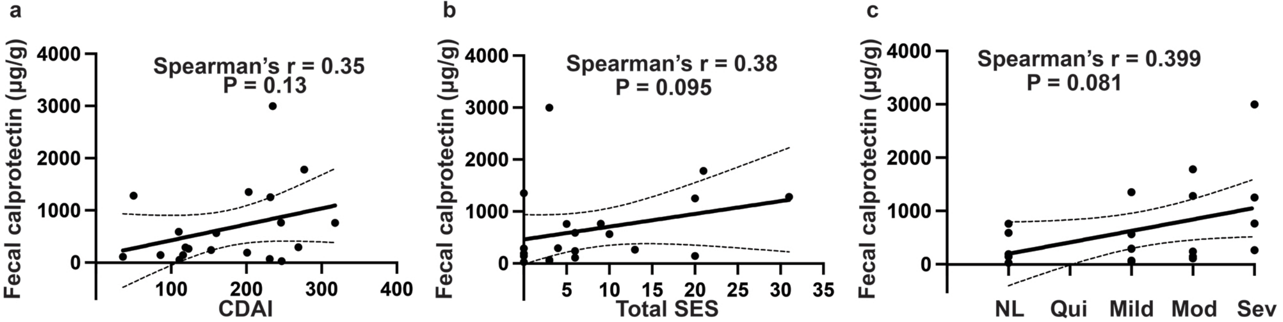

Supplemental Fig S1. Fecal calprotectin is not significantly correlated with disease activity indices. Using the sub-cohort of CD patients for which fecal calprotectin was available, (n=20), we assessed a) CDAI vs. fecal calprotectin, b) SES vs. fecal calprotectin, and c) Histologic severity vs. fecal calprotectin using Spearman's correlation. NL = normal, Qui = quiescent, Mod = moderate, and Sev = severe. r = rho.
